# Immunoaffinity Plastic Blade Spray Mass Spectrometry for Rapid Confirmatory Analysis of Food Contaminants

**DOI:** 10.1021/jasms.2c00149

**Published:** 2022-10-12

**Authors:** Ariadni Geballa-Koukoula, Arjen Gerssen, Marco H. Blokland, Michel W. F. Nielen

**Affiliations:** † Wageningen Food Safety Research, Wageningen University & Research, P.O. Box 230, 6700 AE Wageningen, The Netherlands; ‡ Laboratory of Organic Chemistry, Wageningen University, Stippeneng 4, 6708 WE Wageningen, The Netherlands

## Abstract

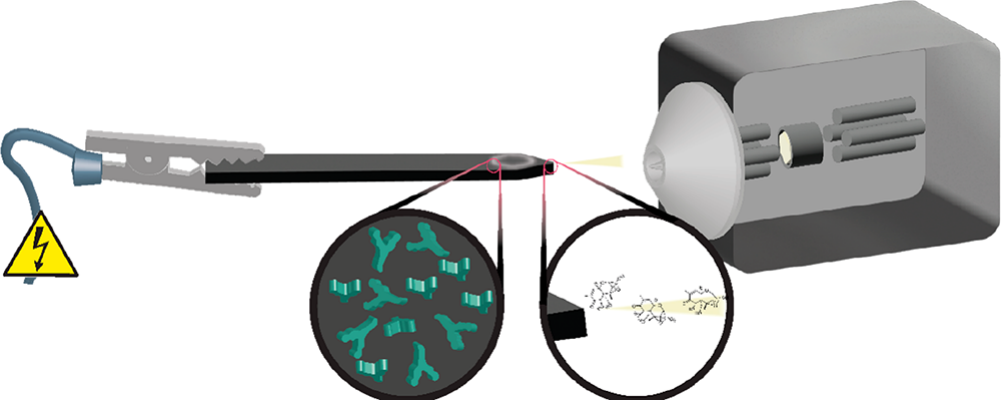

The lack of chromatographic separation in ambient and direct mass spectrometry (MS) ionization techniques jeopardizes the overall selectivity of the developed methods. Incorporating a biosensing element at the ionization source could compensate for that inherent lack of selectivity. Thus, a simplified immunoaffinity-direct MS technique was developed, immunoaffinity blade spray (iBS), featuring a conductive polystyrene blade material. In iBS, the generic coating used in conventional coated blade spray is replaced with a layer of highly specific monoclonal antibodies (mAbs), while the stainless steel is replaced with conductive polystyrene to allow for simple ELISA platelike hydrophobic immobilization of mAbs. Because of its high relevance for climate change-induced food safety issues, the mycotoxin deoxynivalenol (DON) was chosen as a model substance. Following a rapid extraction from wheat flour, DON is immuno-captured, and the blade is positioned in front of the MS for direct iBS-MS/MS analysis. The method’s applicability was demonstrated by analyzing spiked and incurred wheat flour samples, omitting the need for time-consuming chromatographic separation. Apart from DON, cross-reacting DON conjugates could be successfully analyzed as well. The direct iBS-MS/MS method is generic and adaptable to detecting any analyte in sample extracts, provided that specific mAbs are available.

Coated blade spray (CBS) has emerged as a straightforward ambient mass spectrometry (MS) ionization method, utilizing a sharp-tipped stainless steel sheet coated with a biocompatible solid phase microextraction (SPME) sorbent.^1^ CBS combines sample cleanup and ionization directly from the same surface; the blade acts as a solid-substrate electrospray ionization (ESI) source and the coating as an extraction/preconcentration agent. Ionization in CBS occurs by applying a desorption/spray solution and a high voltage.^2^ CBS allows high-throughput analysis and reduced matrix effects in complex sample mixtures analyses while consuming minimal sample and solvent volumes, thus contributing to a greener analytical chemistry.^3^ In only six years since its development in 2014, various applications of CBS in different fields have been reported in the literature.^4−6^ A typical CBS protocol consists of preconditioning the coated surface, extracting the analytes of interest from the sample, washing to minimize interference, and desorption/ionization, all from the same blade.^2^ This simplified protocol leads to an analysis time that can be as short as 10 s per sample,^7^ which in comparison to the classic approach of liquid or gas chromatography to separate the analytes in time^8,9^ is a great improvement for a prompt analytical response. Nonetheless, when it comes to the unequivocal identification of substances, apart from the selective MRM transitions from a tandem MS method, also the retention time acts as an identification criterion,^10,11^ especially in terms of food safety regulation.^12^ Still, the retention time is lacking, by definition, in all ambient and direct ionization techniques.^13^

To counterbalance the lack of chromatographic separation, antibodies have been employed for specific extraction/separation of the targeted analyte, followed by ambient or direct ionization and fast MS identification.^14,15^ The latest development combines CBS with antibody enrichment in the developed immuno-enriched paramagnetic microspheres–magnetic blade spray (iMBS), in which the coating of the blades has been replaced by mAbs-enriched paramagnetic microspheres, held on the blade by a magnet.^16^ This method has demonstrated the added specificity and selectivity in the direct MS/MS analysis of analytes in complex sample matrices. However, the paramagnetic microspheres and the magnet increase the cost and complexity of the analysis: iMBS requires a rather extensive protocol for microsphere and blade preparation based on a covalent immobilization protocol entailing an EDC/NHS amine coupling procedure.^17^ Generally, in immunoassays, the choice of the immobilization strategy is greatly affected by the physicochemical properties of the surface and the antibodies, and physical immobilization can be a simple alternative to circumvent time-consuming covalent coupling reactions. Physical immobilization includes direct adsorption on a surface, and despite the weak attachment and random orientation of antibodies, it is easier and more simple compared to other immobilization methods.^18^ Physical adsorption on the solid polystyrene (i.e., plastic) substrate by hydrophobic interactions has been employed traditionally in enzyme-linked immunosorbent assay (ELISA).^19^ In such a case, the biorecognition element is diluted in a coating buffer and then deposited on the polystyrene substrate while incubating to enable immobilization, without any additional reagents needed.^20^

In the current study, we develop an immunoaffinity conductive polystyrene blade spray (iBS) method. iBS utilizes conductive polystyrene sheets shaped at the dimensions of the stainless steel blades used in CBS. Previous research on CBS applications that employed different substrates, i.e., magnetic blades, required an adhesive copper tape for electric conductivity.^21^ However, the conductive polystyrene allows for simplified mAbs immobilization by adsorption while enabling ionization. The mAbs allow for the selective mining of a targeted analyte, adding selectivity and specificity to the overall direct MS/MS method. The sample extract is incubated with the immunoaffinity blades, followed by washing to remove nonspecifically bound analytes. The final step is the direct spray ionization by applying the optimized dissociation/spraying solution and the high voltage.

The method was developed to detect the mycotoxin deoxynivalenol (DON) as a proof-of-principle. DON is found in *Fusarium* sp. contaminated cereals, and DON’s presence in food commodities risks human health. Consumption of DON-contaminated food could include vomiting (thus why DON is also named vomitoxin), diarrhea, abdominal pain, headache and dizziness.^22^ For this reason, DON is strictly monitored in the EU, with a maximum level (ML) of 1750 μg/kg in unprocessed durum wheat.^23^ Moreover, DON’s societal relevance is eminent because of the climate change-related increase in worldwide mycotoxin production, which issues the increased need for monitoring in the near future.^24,25^ DON is thermally stable and water-soluble,^22^ but its presence in its conjugated/masked forms due to plant metabolism makes its analytical detection elaborate. The developed iBS method can specifically and reproducibly monitor DON and its conjugated form of 3-acetyldeoxynivalenol (3-AcDON) due to the selectivity of the employed mAb targeting only DON and conjugates.^26^ iBS is the first approach for a simplified direct immuno-capture followed by blade spray ionization, all from the same solid surface, without intricate chemical antibody immobilization.

## Experimental Section

### Chemicals and Materials

**Figure 1:**
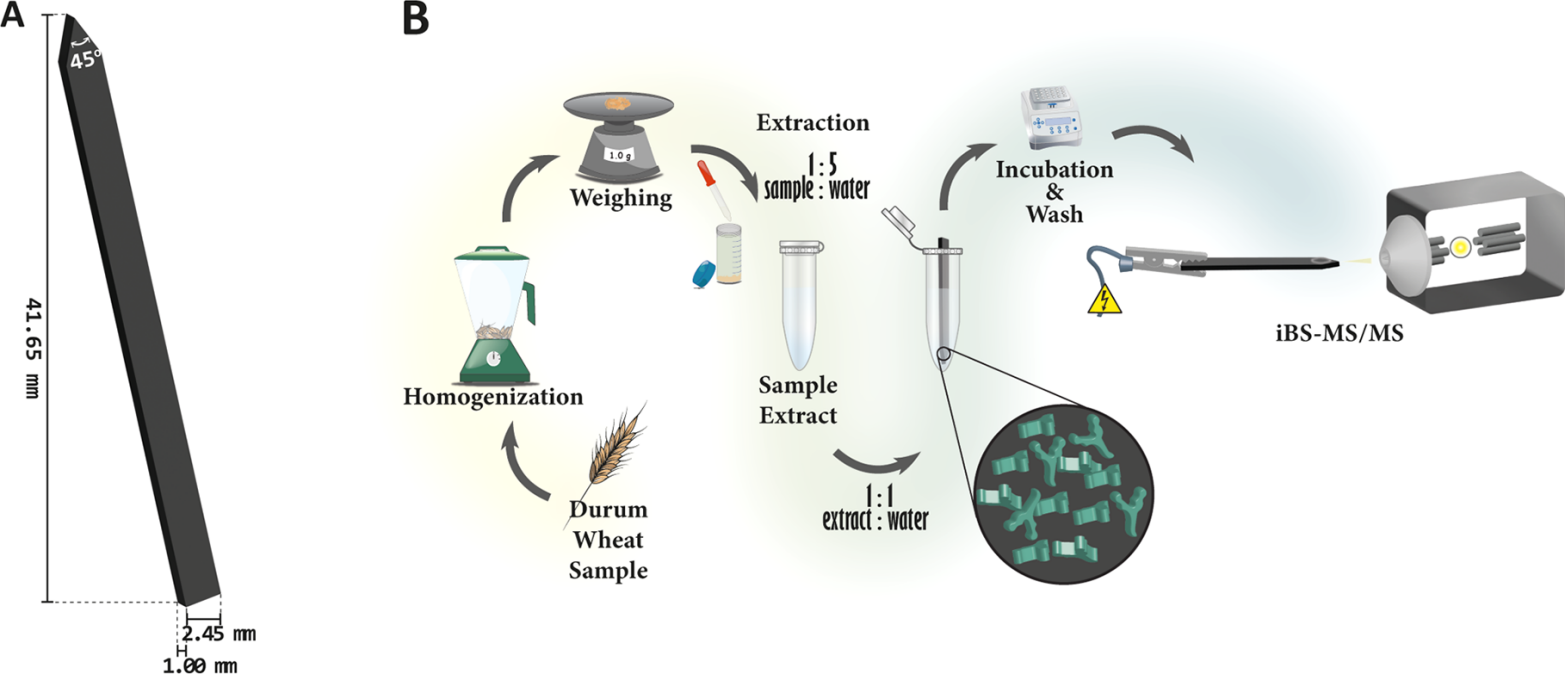
(A) Detailed illustration and dimensions of the conductive polystyrene blade. (B) General concept of the iBS-MS/MS approach.

Conductive polystyrene blades were prepared by laser-cutting conductive polystyrene sheets of 1 mm thickness (Merck, Darmstadt, Germany) at the detailed dimensions of Figure 1A. For the immuno-capturing, monoclonal antibodies for DON (mouse, clone 2) (Aokin AG, Berlin, Germany) were used.

Solvents purchased included acetonitrile, methanol, and water, all of UHPLC-MS purity grade, and ammonia solution 25% v/v, formic acid 98%v/v, from Merck (Darmstadt, Germany), while Milli-Q water of 18.3 MΩ/cm conductivity was produced by a water purification system from Merck (Amsterdam, The Netherlands). A stock solution of 10× phosphate-buffered saline (PBS) with salts from Merck (Darmstadt, Germany) was prepared in Milli-Q water. Dilution of the stock solution to 1× PBS in Milli-Q water and 0.05% v/v Tween-20 (Sigma-Aldrich, Zwijndrecht, The Netherlands) or 1% bovine serum albumin (BSA) (Sigma-Aldrich, Zwijndrecht, The Netherlands) yielded the assay buffers PBST and PBS-BSA, respectively.

Standard stock solutions of 100 μg/mL of DON and 3-AcDON and 25 μg/mL of ^13^C-DON internal standard (IS) and DON blank wheat flour certified reference material (CRM) (Joint Research Centre) were all purchased from LGC Standards (Wesel, Germany). Fluorescent labeled fluorescein-DON was purchased from Aokin (Berlin, Germany). A naturally incurred wheat flour (2900 μg/kg) sample was purchased from Trilogy Analytical Laboratories (Arnhem, The Netherlands).

### Instrumentation

The iBS-MS/MS analysis was performed with a Micromass Quattro Ultima Pt QqQ-MS system (Waters Corporation, Milford, MA) equipped with a blade spray setup consisting of a modified *x*-*y*-*z* stage and high-voltage plug from a Waters nanoESI ion source. Chronograms were acquired in positive ionization mode in multiple reaction monitoring (MRM) mode, and two transitions were monitored for each analyte; for DON, *m*/*z* 297.1 > 231.1 and *m*/*z* 297.1 > 249.1 at 10 and 8 eV collision energy respectively, for 3-AcDON, *m*/*z* 339.10 > 203.10 and *m*/*z* 339.10 > 231.10 at 10 and 8 eV collision energy, respectively, and ^13^C-DON 312.10 > 263.10, at 8 eV collision energy, respectively. From the two fragments ions, ion ratios were calculated and used for unequivocal identification of each substance according to the EU criteria for confirmatory analytical methods.^12^ Operating conditions included 4.2 kV spray voltage, 50 V cone voltage, 120 °C cone temperature, and 0.16 mL/min argon collision gas flow. MassLynx software (Waters) was used for data acquisition and processing. A Voltcraft 7910 multimeter was used for conductivity measurements, and two microscopes, namely an Olympus BX51 fluorescence microscope and a Dino-lite AM4115T-GFBW digital microscope, were used for fluorescence imaging.

### Methods

Using Zeba Spin desalting columns (Thermo Fisher Scientific, San Jose, CA), the storage buffer of the crude mAbs from Aokin is removed, and the mAbs are reconstituted in UHPLC–MS purity grade water. The mAbs are then diluted with UHPLC–MS purity grade water to a 0.3 mg/mL final concentration. Finally, 7.5 μL is simply pipetted on top of the conductive polystyrene blade and nearest to the sharp tip, and the prepared immunoaffinity blades are left to air-dry and are stored in the refrigerator at 4 °C until further use.

The simplified extraction method described previously for DON was applied without further optimization.^26^ Briefly, 1 g of ground wheat sample is extracted using 5 mL Milli-Q water. Following manual agitation and centrifugation to fasten sedimentation, the supernatant is collected and used in the case of incurred wheat or, in the case of blank wheat, spiked at 350 ng/mL (corresponding to the DON concentration in the extract following extraction of contaminated commodities at the ML of 1750 μg/kg). For iBS analysis, 200 μL of the sample extract is diluted 1:1 Milli-Q water, placed in an Eppendorf tube with a single immunoaffinity blade, and incubated for 2 min. Next, the immunoaffinity blade is washed with 500 μL of Milli-Q water for 30 s. Both immuno-extraction and washing are performed in an Eppendorf ThermoMixer C apparatus (Eppendorf SE, Hamburg, Germany) at 1200 rpm. For the iBS-MS/MS detection, the immunoaffinity blade is positioned at approximately 6 mm distance from the ion source cone. Then, the optimized dissociation/spray solution is pipetted on top of the mAbs of the blade twice. First, 4 μL of methanol/ammonia solution 2% v/v is pipetted to disrupt the binding between DON and mAbs. Second, after evaporation of the first aliquot, an additional 4 μL of methanol/ammonia solution 2% v/v spiked with the internal standard (IS) ^13^C-DON 10 ng/mL is pipetted, and the optimum spray voltage is applied to generate an ESI-like spray (Figure 1B).

## Results and Discussion

### Preparation and Characterization of the Immunoaffinity Blades

To produce the immunoaffinity blades, a large conductive polystyrene sheet was laser-cut in the desired size and shape (Figure 1A). As discussed in previous CBS and iMBS studies, the angle of the blade is crucial for the ionization of substances; the tip of the blade is where the applied voltage is converged, leading to an ESI-like spray formation.^1^ Thus, the conductive polystyrene blades’ selected shape is characterized by a pointed tip with an adequate surface for handling, mAbs immobilization, solvent deposition, and electrospray formation. After shaping the conductive polystyrene blades, mAbs for DON must be immobilized on the blade’s tip. The mAbs are stored in buffer solution containing surfactants and salts that hinder ionization in MS, and their residues in the immunoaffinity blade could pose a risk for the success of the iBS-MS/MS method. Thus, the mAbs, prior to the immobilization, underwent buffer exchange using size-exclusion chromatography resin in desalting Zeba Spin columns to remove the excess buffer and reconstitute them in pure water. Then the mAbs were diluted to the desired concentration, pipetted on the conductive polystyrene blade’s tip, and immobilized on the surface after drying, simply by direct adsorption.

Unsurprisingly, the conductive polystyrene blades are characterized by an electric conductance 188,000 times lower than the standard stainless steel blades. Nevertheless, the main voltage drop between the voltage application point and the inlet of the MS still occurs in the ambient air gap between the blade tip and the cone. The lower conductance is also apparent when comparing a standard solution of 10 ng/mL DON, 3-AcDON, and IS in methanol/ammonia solution 2% v/v on the different blade materials. At 3.7 kV spray voltage, while the response factor (analyte/internal standard area ratio - A/IS area ratio) is identical between conductive polystyrene and stainless steel blades, the absolute area values in the MS/MS chronograms drop by 94%. Therefore, the high voltage setting had to be optimized for conductive polystyrene and was found to be 4.2 kV. It is worth mentioning that even with the optimum spray voltage for DON ionization on conductive polystyrene blades the conductive polystyrene blades still yielded 65% lower ionization than the stainless steel blades at 3.7 kV (Figure S1). Most likely, this difference must be attributed to the different geometries of the blades. The tip angles are similar, but the thickness of the metal and polystyrene sheets differ by a factor of 10, so the polystyrene blade required a further manual adjustment to 0.1 mm with a scalpel.

Based on the overall sensitivity of the iBS-MS/MS method, the mAbs maximum theoretical loading capacity, and the regulatory limits for DON monitoring, a limited volume of 7.5 μL 0.3 mg/mL mAbs solution per blade was fit-for-purpose, similar to the mAbs consumption in the identification lateral flow immunoassay (ID-LFIA) direct MS alternative approach.^27^

**Figure 2:**
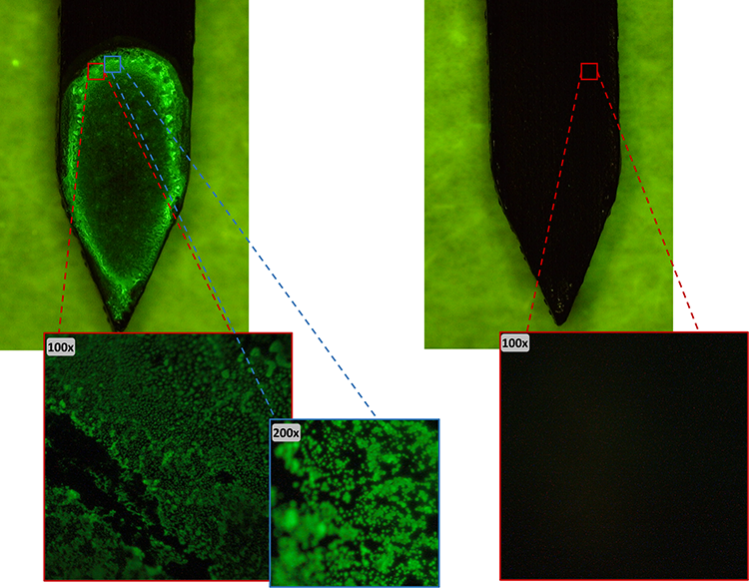
Fluorescence imaging with excitation wavelength of 460–490 nm and emission of 510–550 nm (before and after cleaning) of the immunoaffinity blade’s tip after adding fluorescein-DON, followed by washing with water. In the insets, 100× and 200× magnifications are depicted.

Fluorescence imaging was used to verify the coating at the immuno-enriched area on the conductive polystyrene blade. Five μL of fluorescein-DON was first deposited on the immunoaffinity blade and washed with Milli-Q water to remove the nonbound analyte, followed by the excitation and fluorescent imaging. As expected, only the immuno-enriched area of the blade was fluorescent. Moreover, the fluorescence intensity was increased at the edge of the immuno-enriched area suggesting a higher concentration of the mAbs; a typical characteristic of the so-called coffee-ring effect after evaporation of liquid from the center to the edge^28^ (Figure 2). Using 200× magnification, the surface of the conductive blade was observed to be not homogeneously coated but instead consisting of mAbs aggregations, resulting from the simplified but uncontrolled physical immobilization of antibodies. Finally, the immunoaffinity blades were cleaned by sonicating in methanol for 15 min and wiping the surface to remove the mAbs. The cleaning resulted in bare conductive polystyrene blades that could be reused for immuno-enrichment in other iBS experiments. The cleaning performance was demonstrated using the same process of fluorescein-DON addition, water washing, and fluorescence imaging. No fluorescence is observed on the cleaned blade, which demonstrates the complete removal of mAbs and the effectiveness of the washing procedure for the removal of unbound analytes (Figure 2).

### iBS-MS/MS Method Development and Application

The MS method optimization was performed at a distance of approximately 6 mm between the tip of the blade and the inlet of the MS; larger distances caused signal loss, while at a reduced distance, arcing occurred. The optimum spray/desorption solution was selected by applying on the conductive polystyrene blades 5 μL of 200 ng/mL DON in various solutions and monitoring the area in the chronograms for the protonated and deprotonated ions in full scan mode (*m*/*z* 250–500) under different spray voltage settings in positive- and negative-ion mode. The solvents were selected to include a high percentage of organic solvent and alkaline or acidic modifier. The organic solvent reassures high ionization efficiency in the blade spray part, and the modifier supports the dissociation of the analyte from the antibodies in the final iBS-MS/MS method. The optimized spray solution was similar to previously published results,^27^ i.e., methanol/ammonia solution 2% v/v (Figure S1C). However, regardless of the solvent used, the negative ionization mode was not as efficient as positive ionization, contrary to previously published results;^27^ clearly, the different MS systems account for these differences. The optimum cone voltage and collision energy were investigated by applying 5 μL of 200 ng/mL DON in the optimum solution, methanol/ammonia solution 2% v/v. For the cone voltage, values were varied from 20 to 110 V with a step of 10 V. Minor differences were observed in the area of the protonated ion of DON at different cone voltages settings; thus, the selected cone voltage was 50 V. Furthermore, the collision energy was optimized starting from 2 to 20 eV, with a step of 2 eV, and monitoring the area of the main fragment ions. The optimized MRM transitions were *m*/*z* 297.1 > 231.1 at 10 eV and *m*/*z* 297.1 > 249.1 at 8 eV collision energy for DON, *m*/*z* 339.10 > 203.10 at 10 eV and *m*/*z* 339.10 > 231.10 at 8 eV collision energy for 3-AcDON, and *m*/*z* 312.10 > 263.10 at 8 eV collision energy for the IS ^13^C-DON.

**Figure 3:**
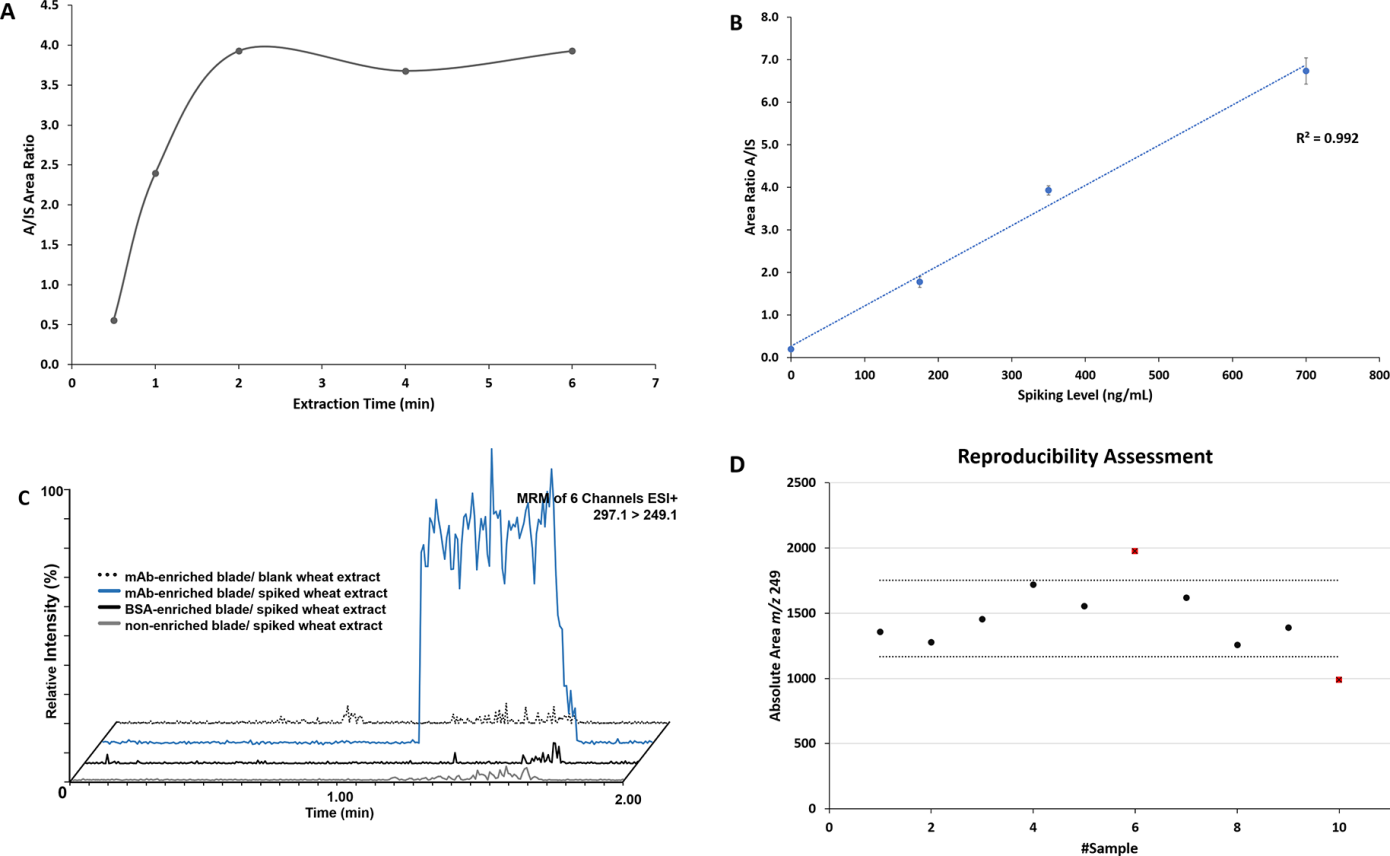
iBS-MS/MS method development and performance. (A) Extraction by immuno-capturing–time optimization (B) Quantitative analysis calibration curve. (C) Feasibility of the iBS-MS/MS method. Overlay chronograms of the *m*/*z* 297.1 > 249.1 transition obtained following the iBS-MS/MS extraction, immuno-capture, and ionization protocol using mAbs-enriched, BSA-enriched, and nonenriched blades. (D) Reproducibility of the iBS-MS/MS method. Results within the acceptable 20% RSD% are within the dotted lines, except for blades #6 and #10 which are outliers. For detailed conditions and procedures, see text.

For the iBS protocol development, the approach was adopted from standard CBS methods, composed of (i) conditioning of the blades; (ii) extraction/immunocapturing of the targeted analyte from the sample or sample extract; (iii), rinsing or washing the surface to remove interfering species; and (iv) the desorption/ionization of the analytes from the blade.^2^ The blade’s conditioning step was examined as part of the extraction step by using different buffers and Milli-Q water in 1:1 ratio with the 350 ng/mL DON spiked blank wheat extract and monitoring the A/IS area ratio in the chronograms. Although assay buffers can be used to promote interactions between analytes and mAbs, the calculated A/IS area ratio revealed that 1 × PBS-T (0.05% Tween-20) resulted in a 10% decrease in the mean area, and 1 × PBS-1% BSA resulted in a 14% decrease in the mean area compared to Milli-Q water. The decrease in the mean area may result from some buffer residues leading to ion suppression. For the sample extraction, different undiluted volumes of sample extracts were tested, namely, 200, 400, and 600 μL of 350 ng/mL DON spiked wheat extract. There was a relative decrease in the A/IS area ratio with the volume increase. So, 200 μL of sample extract was optimum for the extraction process (Figure S2A). Further, a comparison was made between 200 μL of DON spiked undiluted wheat extract at 350 ng/mL and 200 μL of DON spiked wheat extract at 350 ng/mL diluted 1:1 with Milli-Q water. In this case, the mean area was 50% decreased in the undiluted sampling, possibly due to matrix interferences hindering the biorecognition and causing ion suppression. For this reason, a 1:1 dilution of the wheat extract with Milli-Q water was chosen in the final protocol. Furthermore, the incubation time, i.e., the time of the immuno-capturing, was evaluated by assessing different incubation times from DON spiked sample extract at 350 ng/mL at 1:1 dilution with Milli-Q water and plotting them against the A/IS chronogram area ratio obtained from the iBS-MS/MS analysis. The incubation was performed in an Eppendorf Thermomixer at room temperature and 1200 rpm to reassure the reproducibility of the procedure (cf. the Experimental Section). After 2 min of incubation, a plateau was reached due to a limited capacity of the antibodies for the immuno-capturing to reach an equilibrium. Therefore, 2 min is used in the optimized extraction protocol (Figure 3A). Finally, various washing solution compositions and washing durations were tested for washing optimization, namely 500 μL of methanol/Milli-Q water in 0/100, 20/80, 50/50, and 80/20% v/v and 10, 30, and 60 s with 500 μL of Milli-Q water. A high percentage of organic solvent promotes denaturation of the mAbs and untimely dissociation of the analyte from the mAbs. As expected, the A/IS area ratio of 3.7 with 0% methanol decreased by 37.8%, 94.3%, and 97.3%, with increasing percentages of methanol. Concerning the washing duration, 30 s produced the optimum result, which can be concluded as enough time to remove nonspecifically bound analyte and remove matrix components from the wheat extract following immuno-capturing. For that reason, 30 s was selected as the most efficient washing time (Figure S2B).

Next, the quantitative performance of the optimized protocol in the relevant range was evaluated by spiking blank wheat extract with DON at three different target levels (TL). Two individual plastic blades were used to analyze the same sample extract. The TL were based on the ML of 1750 μg/kg for DON in unprocessed durum wheat to a final level of 175 ng/mL (0.5 × TL), 350 ng/mL (TL), and 700 ng/mL (2 × TL). Good linear regression of 0.992 was observed (Figure 3B).

The desorption step spray performance of iBS was examined using the optimized spray solution. Four μL was found to be the optimum applied volume when it comes to ionization from the iBS, as it creates a stable ESI-like spray, and also it does not elongate the ionization process beyond 50 s, as the 5 μL used for method development did. The methanol/ammonia solution 2% v/v has been examined in a previous publication to disrupt the binding of DON from the mAbs in surface plasmon resonance (SPR).^27^ Due to differences in the surfaces between the blade and the SPR chip, the latter being in constant liquid flow contact, methanol/ammonia solution 2% v/v did not quantitatively dissociate the entirety of bound DON at once. Multidesorption steps (6 in total for a sample of 350 ng/mL) are required for a complete desorption/ionization of the analyte bound with the mAbs. Despite that, the first single desorption already provides sufficient chronogram area counts for quantitation in a reproducible manner, so multidesorption steps were superfluous (Figure S2C).

To illustrate the feasibility of the iBS-MS/MS approach, 200 μL of blank wheat extract was spiked at 350 ng/mL, diluted 1:1 with Milli-Q water, and incubated for 2 min with (a) an immuno-affinity blade (mAbs-enriched), (b) an BSA-enriched blade, and (c) a bare conductive polystyrene blade. In addition, the mAbs-enriched blade was incubated with unspiked blank wheat extract and diluted 1:1 with Milli-Q water. After 30 s of washing of the blades with 500 μL of Milli-Q water and MS/MS analysis, the results demonstrated, as anticipated, a positive signal for DON with ion ratio for *m*/*z* 231.1/249.1 of 0.42 originated only from the immuno-affinity blade incubated with spiked sample extract and from none of the other blades used. The ion ratio falls within the tolerance limit of the EU criteria for confirmatory methods^12^ for the ion ratio, since the ion ratio *m*/*z* 231.1/249.1 for DON in standard solutions was 0.47. This clearly demonstrates the added value of the mAbs for selective immuno-capturing and extraction since the positive signal resulted from the immuno-affinity blades and not from DON adsorbed on the bare conductive polystyrene surface (Figure 3C).

Further, it was noticed that after repeated use the polystyrene blades start to show differences in the tip’s sharpness, probably because of the extraction step performed with the blades’ tip facing downward and pressing against the bottom of the Eppendorf tube. For this reason, the reproducibility of the immunoaffinity blades was assessed by analyzing 10 individual blades following DON immuno-capturing from the same wheat extract spiked at 350 ng/mL. Results of the absolute area values of the MS/MS chronograms had a ±22% RSD, which is above the permitted by the EU regulation.^12^ Nonetheless, the corrected value used, i.e., the A/IS chronogram area ratio, was within ±4% RSD for all the 10 blades analyzed. As in many ambient and direct MS methods, an IS during ionization is necessary for reproducibility. However, for a quick qualitative confirmation of identity, also the absolute areas yielded a positive result with a stable ion ratio regardless (Figure 3D and Table 1).

**Table 1:** Reproducibility Assessment of 10 Individual Immunoaffinity Blades Using iBS-MS/MS Analysis^a^

	absolute area values				
	DON			^13^C DON	
immunoaffinity blade no.	*m*/*z* 297.1 > 249.1	*m*/*z* 297.1 > 231.1	ion ratio	*m*/*z* 312.1 > 263.1	response factor (A/IS)
#01	1358	603	0.44	339	4.00
#02	1280	560	0.44	305	4.19
#03	1455	605	0.42	376	3.87
#04	1719	717	0.42	448	3.84
#05	1556	740	0.48	396	3.93
#06	1974	906	0.46	482	4.10
#07	1622	675	0.42	380	4.27
#08	1257	594	0.47	301	4.18
#09	1392	597	0.43	354	3.93
#10	990	386	0.39	242	4.09
					RSD 4%

a Conditions: single measurement of 10 individual immunoaffinity blades, following extraction with immuno-capture, wash, and iBS-MS/MS analysis. RSD is the relative standard deviation of the ten measurements. The ion ratio is the area ratio of the two ion transitions for DON, *m*/*z* 231.1/249.1.

**Figure 4:**
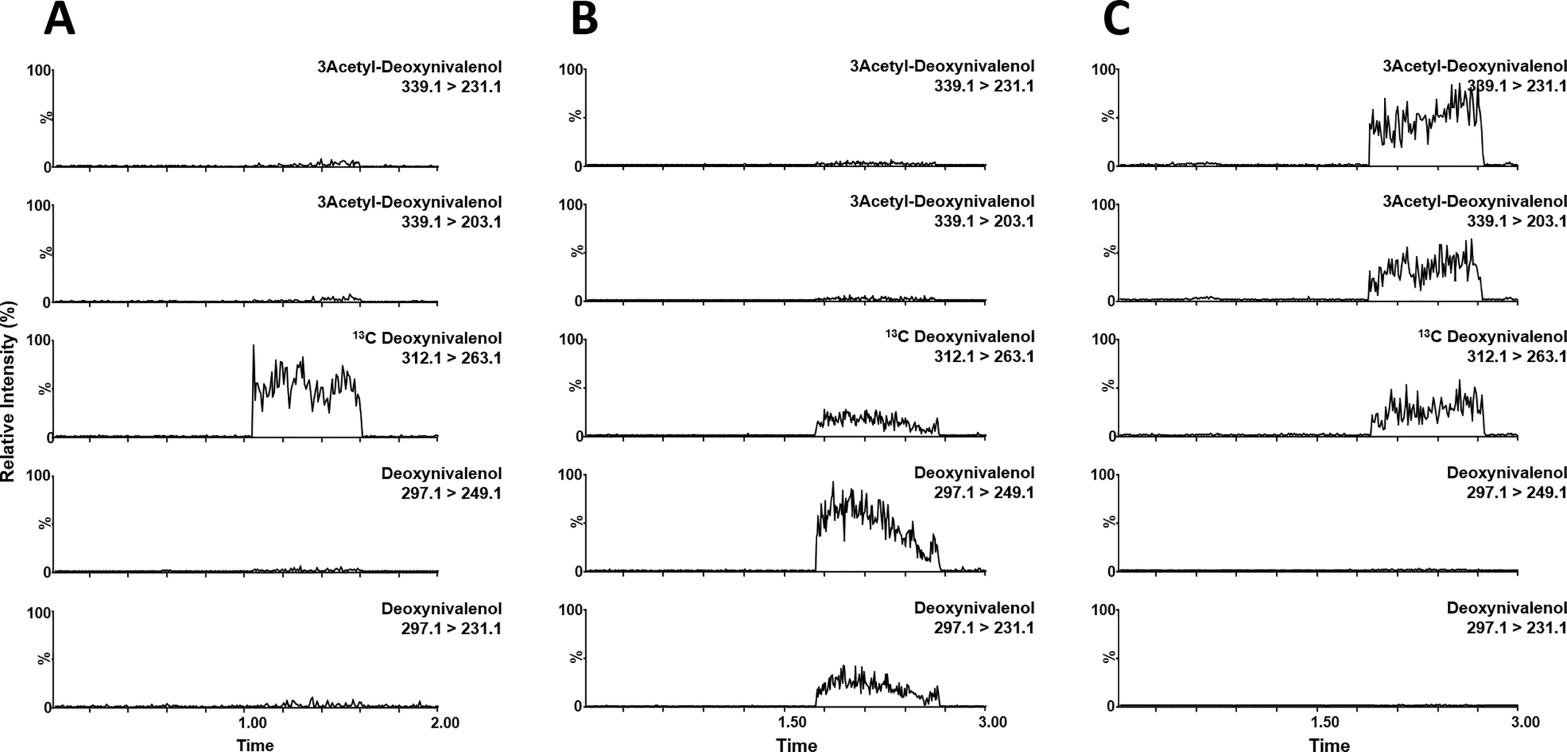
Representative chronograms from the analysis of (A) blank wheat, (B) incurred wheat, and (C) 3-AcDON spiked wheat. The intensities are normalized on based on the highest intensity of each individual chronogram. For the exact conditions of the iBS-MS/MS analysis, see the text.

Finally, the applicability of the optimized method described in Figure 1 was illustrated by the analysis of different additional spiked and incurred samples. The samples included a blank CRM wheat flour, the same extract but spiked with 3Ac-DON at 175 ng/mL (TL for DON), and a 2900 μg/kg (≈ 1.6 × ML for DON) incurred wheat sample (Figure 4). The mean ion ratio for *m*/*z* 203.1/231.1 of 3-AcDON was 0.62 (±0.04), a ratio identical to that of the standard solution of 3-AcDON in methanol/ammonia solution 2%v/v and within the regulatory EU criterion of 20% RSD. Moreover, the response factor A/IS for the chronogram area of 3-AcDON versus ^13^C-DON was 3.1 (±0.1) for two individual measurements of iBS-MS/MS, thereby clearly differentiating the spiked from the blank CRM wheat sample. For the contaminated wheat sample, iBS-MS/MS results showed an A/IS area ratio for DON of 4.4 (±0.4), corresponding to a quantitative result of 535.6 (±14.2) ng/mL, for two individual iBS-MS/MS measurements, calculated from the calibration curve and pointing to a level of 2678 μg/kg in the contaminated wheat sample analyzed.

## Conclusions

Combining antibodies with direct MS analysis is an obvious advantage in raising the specificity of a rapid MS method. In this work, a simplified iBS-MS/MS method was presented, exploiting, first, the ease of antibody adsorption on polystyrene surfaces, second, the commercial availability of conductive polystyrene, and third, direct MS ionization. iBS-MS/MS is generic, enables semiquantitative and reproducible analysis, and can be used for a fast, more secure screening or confirmation of substances, given the high specificity of mAb. iBS-MS/MS results suggest that the mAb activity is not compromised on the polystyrene blade and mAb leads to a selective immuno-extraction. Further, iBS-MS/MS highlights the opportunity to use alternative conductive surfaces for direct MS approaches. For instance, investigating conductive surfaces with increased strength could eliminate the observed mechanical deformation that leads to unsteady ESI-like spraying. The conductive polystyrene blades can be cleaned to remove the mAbs and reused (up to 9 times) following immobilization of new mAbs leading to an eco-friendlier analytical approach. Moreover, it can straightforwardly confirm the identity of the analyte bound on the mAbs of the blade, with a total time from sample to MS analysis that does not exceed 5 min, leading to high-throughput analysis in the case where a respective autosampler is used, and a total cost of 2.5 euros per iBS-MS/MS analysis, which could vary of course depending on the cost of the mAb. Theoretically, apart from DON, iBS-MS/MS can be adapted to detect any other low molecular weight analyte in a similar hyphenation approach, provided that mAbs are available, also paving the road to multiplex iBS-MS/MS opportunities.

## Supporting Information Available

Comparison between conductive polystyrene and stainless steel blades and spray voltage optimization (Figure S1); iBS-MS/MS method development (Figure S2) (PDF)

## References

[ref1] Gómez-Ríos G. A.; Pawliszyn J. Development of Coated Blade Spray Ionization Mass Spectrometry for the Quantitation of Target Analytes Present in Complex Matrices. Angew. Chemie - Int. Ed 2014, 53 (52), 14503–14507. 10.1002/anie.201407057. 25384839

[ref2] Gómez-Ríos G. A.; Tascon M.; Pawliszyn J. Coated Blade Spray: Shifting the Paradigm of Direct Sample Introduction to MS. Bioanalysis 2018, 10, 257–271. 10.4155/bio-2017-0153. 29376744

[ref3] Gómez-Ríos G. A.; Tascon M.; Reyes-Garcés N.; Boyacl E.; Poole J.; Pawliszyn J. Quantitative Analysis of Biofluid Spots by Coated Blade Spray Mass Spectrometry, a New Approach to Rapid Screening. Sci. Rep 2017, 7 (1), 16104 10.1038/s41598-017-16494-z. 29170449 PMC5701014

[ref4] Kasperkiewicz A.; Pawliszyn J. Multiresidue Pesticide Quantitation in Multiple Fruit Matrices via Automated Coated Blade Spray and Liquid Chromatography Coupled to Triple Quadrupole Mass Spectrometry. Food Chem. 2021, 339, 127815 10.1016/j.foodchem.2020.127815. 32836024

[ref5] Khaled A.; Gómez-Ríos G. A.; Pawliszyn J. Optimization of Coated Blade Spray for Rapid Screening and Quantitation of 105 Veterinary Drugs in Biological Tissue Samples. Anal. Chem. 2020, 92 (8), 5937–5943. 10.1021/acs.analchem.0c00093. 32192344

[ref6] Poole J. J.; Gómez-Ríos G. A.; Boyaci E.; Reyes-Garcés N.; Pawliszyn J. Rapid and Concomitant Analysis of Pharmaceuticals in Treated Wastewater by Coated Blade Spray Mass Spectrometry. Environ. Sci. Technol. 2017, 51 (21), 12566–12572. 10.1021/acs.est.7b03867. 28990769

[ref7] Kasperkiewicz A.; Gómez-Ríos G. A.; Hein D.; Pawliszyn J. Breaching the 10 Second Barrier of Total Analysis Time for Complex Matrices via Automated Coated Blade Spray. Anal. Chem. 2019, 91 (20), 13039–13046. 10.1021/acs.analchem.9b03225. 31429256

[ref8] Vendl O.; Berthiller F.; Crews C.; Krska R. Simultaneous Determination of Deoxynivalenol, Zearalenone, and Their Major Masked Metabolites in Cereal-Based Food by LC-MS-MS. Anal. Bioanal. Chem. 2009, 395 (5), 1347–1354. 10.1007/s00216-009-2873-y. 19572123

[ref9] Cao M.; Li Q.; Zhang Y.; Wang J.; Zhai H.; Ma J.; Sun L.; Wan X.; Tang Y. Determination of Deoxynivalenol and Its Derivative in Corn Flour and Wheat Flour Using Automated On-Line Solid-Phase Extraction Combined with LC–MS/MS. Bull. Environ. Contam. Toxicol. 2021, 107 (2), 248–254. 10.1007/s00128-020-02920-y. 32591852

[ref10] Kuo T. H.; Dutkiewicz E. P.; Pei J.; Hsu C. C. Ambient Ionization Mass Spectrometry Today and Tomorrow: Embracing Challenges and Opportunities. Anal. Chem. 2020, 92, 2353–2363. 10.1021/acs.analchem.9b05454. 31825205

[ref11] Berendsen B. J. A.; Stolker L. A. M.; Nielen M. W. F. The (Un)Certainty of Selectivity in Liquid Chromatography Tandem Mass Spectrometry. J. Am. Soc. Mass Spectrom. 2013, 24 (1), 154–163. 10.1007/s13361-012-0501-0. 23345060

[ref12] Commission Implementing Regulation (EU) 2021/808 of 22 March 2021 on the Performance of Analytical Methods for Residues of Pharmacologically Active Substances Used in Food-Producing Animals and on the Interpretation of Results as Well as on the Methods To. Off. J. Eur. Union 2021, 180, 84–109.

[ref13] Javanshad R.; Venter A. R. Ambient Ionization Mass Spectrometry: Real-Time, Proximal Sample Processing and Ionization. Analytical Methods 2017, 9, 4896–4907. 10.1039/C7AY00948H.

[ref14] Joshi S.; Zuilhof H.; Van Beek T. A.; Nielen M. W. F. Biochip Spray: Simplified Coupling of Surface Plasmon Resonance Biosensing and Mass Spectrometry. Anal. Chem. 2017, 89 (3), 1427–1432. 10.1021/acs.analchem.6b04012. 28208290 PMC5348099

[ref15] Evans-Nguyen K. M.; Hargraves T. L.; Quinto A. N. Immunoaffinity Nanogold Coupled with Direct Analysis in Real Time (DART) Mass Spectrometry for Analytical Toxicology. Anal. Methods 2017, 9 (34), 4954–4957. 10.1039/C7AY00922D.

[ref16] Geballa-Koukoula A.; Gerssen A.; Blokland M. H.; Elliott C. T.; Pawliszyn J.; Nielen M. W. F. Immuno-Enriched Microspheres - Magnetic Blade Spray-Tandem Mass Spectrometry for Domoic Acid in Mussels. Anal. Chem. 2021, 93 (47), 15736–15743. 10.1021/acs.analchem.1c03816. 34726384 PMC8637537

[ref17] Peters J.; Bienenmann-Ploum M.; De Rijk T.; Haasnoot W. Development of a Multiplex Flow Cytometric Microsphere Immunoassay for Mycotoxins and Evaluation of Its Application in Feed. Mycotoxin Res. 2011, 27 (1), 63–72. 10.1007/s12550-010-0077-0. 21836765 PMC3150826

[ref18] Welch N. G.; Scoble J. A.; Muir B. W.; Pigram P. J. Orientation and Characterization of Immobilized Antibodies for Improved Immunoassays (Review). Biointerphases 2017, 12 (2), 02D301 10.1116/1.4978435. 28301944

[ref19] Yu Q.; Wang Q.; Li B.; Lin Q.; Duan Y. Technological Development of Antibody Immobilization for Optical Immunoassays: Progress and Prospects. Critical Reviews in Analytical Chemistry 2015, 45, 62–75. 10.1080/10408347.2014.881249.

[ref20] Pichler H.; Palme S.; Binder E. M.; Krska R. Development of Enzyme-Immunoassays Based on Egg Yolk Antibodies for the Detection of Mycotoxins. Mycotoxin Res. 2001, 17, 202–205. 10.1007/BF03036436. 23605872

[ref21] Rickert D. A.; Singh V.; Thirukumaran M.; Grandy J. J.; Belinato J. R.; Lashgari M.; Pawliszyn J. Comprehensive Analysis of Multiresidue Pesticides from Process Water Obtained from Wastewater Treatment Facilities Using Solid-Phase Microextraction. Environ. Sci. Technol. 2020, 54 (24), 15789–15799. 10.1021/acs.est.0c04875. 33237731

[ref22] Sobrova P.; Adam V.; Vasatkova A.; Beklova M.; Zeman L.; Kizek R. Deoxynivalenol and Its Toxicity. Interdisciplinary Toxicology 2010, 94–99. 10.2478/v10102-010-0019-x. 21217881 PMC2984136

[ref23] European Commission. Commission Regulation (EC) No 149/2008 of 29 January 2008 Amending Regulation (EC) No 396/2005 of the European Parliament and of the Council by Establishing Annexes II, III and IV Setting Maximum Residue Levels for Products Covered by Annex I Thereto. Off. J. Eur. Union 2008, 58, 1–398.

[ref24] Chhaya R. S.; O’Brien J.; Cummins E. Feed to Fork Risk Assessment of Mycotoxins under Climate Change Influences - Recent Developments. Trends Food Sci. Technol. 2021, 10.1016/J.TIFS.2021.07.040.

[ref25] Eskola M.; Kos G.; Elliott C. T.; Hajšlová J.; Mayar S.; Krska R. Worldwide Contamination of Food-Crops with Mycotoxins: Validity of the Widely Cited ‘FAO Estimate’ of 25%. Crit. Rev. Food Sci. Nutr 2020, 60 (16), 2773–2789. 10.1080/10408398.2019.1658570. 31478403

[ref26] Geballa-Koukoula A.; Gerssen A.; Nielen M. W. F. From Smartphone Lateral Flow Immunoassay Screening to Direct MS Analysis: Development and Validation of a Semi-Quantitative Direct Analysis in Real-Time Mass Spectrometric (DART-MS) Approach to the Analysis of Deoxynivalenol. Sensors 2021, 21 (5), 1861 10.3390/s21051861. 33800036 PMC7962121

[ref27] Geballa-Koukoula A.; Gerssen A.; Nielen M. W. F. Direct Analysis of Lateral Flow Immunoassays for Deoxynivalenol Using Electrospray Ionization Mass Spectrometry. Anal. Bioanal. Chem. 2020, 412 (27), 7547–7558. 10.1007/s00216-020-02890-4. 32860092 PMC7533258

[ref28] Mampallil D.; Eral H. B. A Review on Suppression and Utilization of the Coffee-Ring Effect. Adv. Colloid Interface Sci. 2018, 252, 38–54. 10.1016/j.cis.2017.12.008. 29310771

