# Supplementary material for: Immunoaffinity Plastic Blade Spray Mass Spectrometry for Rapid Confirmatory Analysis of Food Contaminants
Source: J Am Soc Mass Spectrom. 2022 Oct 12;33(11):2038–45. doi: 10.1021/jasms.2c00149 (PMC9634800; doi:10.1021/jasms.2c00149)
Supplement: Supplementary file 1 — js2c00149_si_001.pdf [file js2c00149_si_001.pdf]

SUPPORTING INFORMATION

Immunoaffinity Plastic Blade Spray Mass Spectrometry for Rapid Confirmatory Analysis of Food Contaminants

Ariadni Geballa-Koukoula[1], Arjen Gerssen[1], Marco H. Blokland[1*] and Michel W.F. Nielen[2]

[1] Wageningen Food Safety Research, Wageningen University & Research, P.O. Box 230, 6700 AE Wageningen, The Netherlands

[2] Laboratory of Organic Chemistry, Wageningen University, Stippeneng 4, 6708 WE Wageningen, The Netherlands

* Corresponding author email: marco.blokland@wur.nl

Table of Contents

Figure S1. Comparison of conductive polystyrene and stainless steel blades and spray solution optimization S2

Figure S2. iBS-MS/MS method development. S3

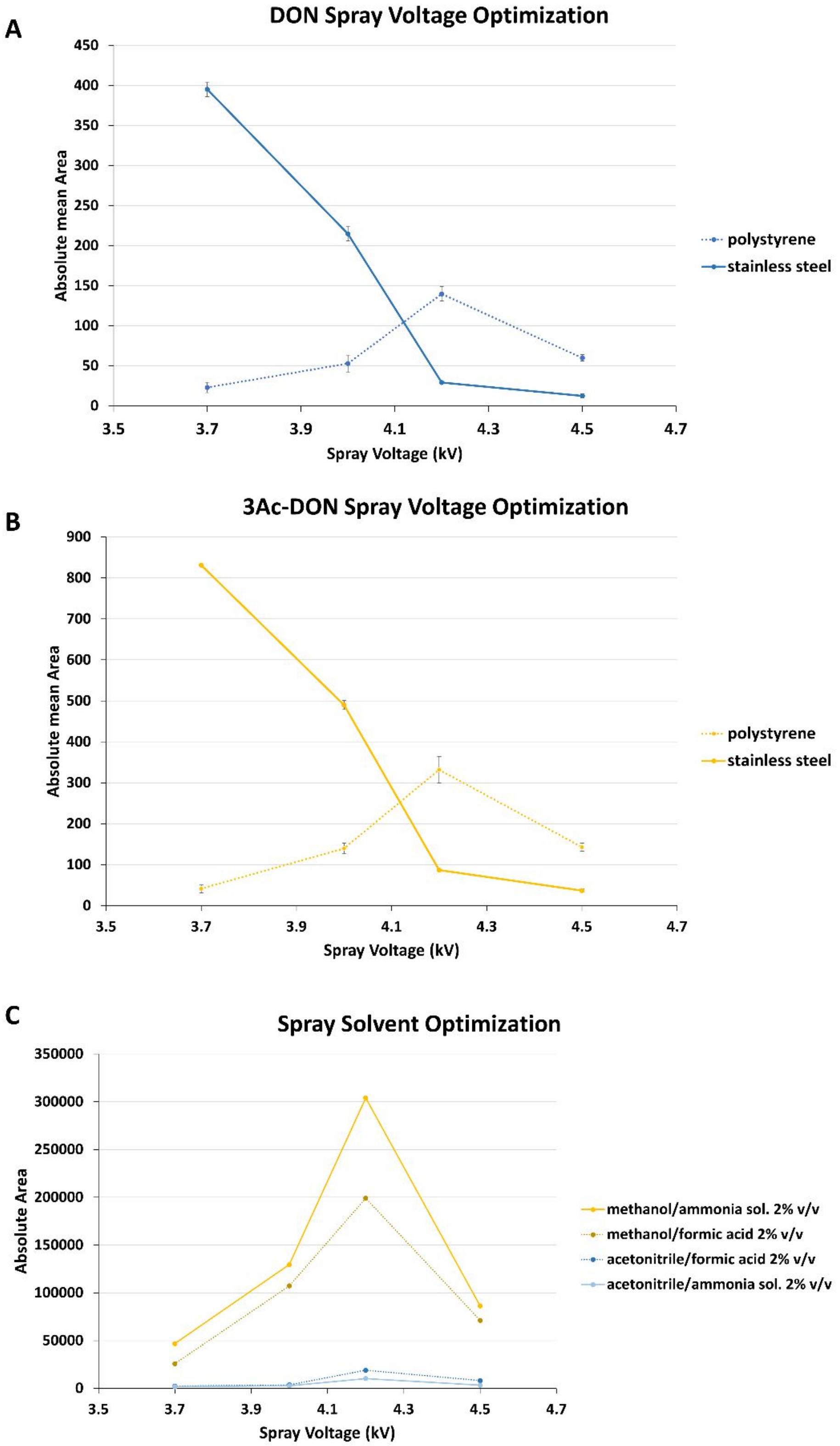

Figure S1. Comparison of conductive polystyrene and stainless steel blades for (A) DON and (B) 3Ac-DON Conditions: 10ng/mL concentration, 5 μL sample application on the blade, MRM monitoring of fragments measuring the area. The comparison is done on different spray voltage intensities and based on the absolute mean values of the area (n=3). (C) Spray solution optimization in different spray voltage values. Conditions: 200 ng/mL concentration, 5 μL sample application, full scan mode monitoring the protonated ion of DON and measuring the area absolute value.

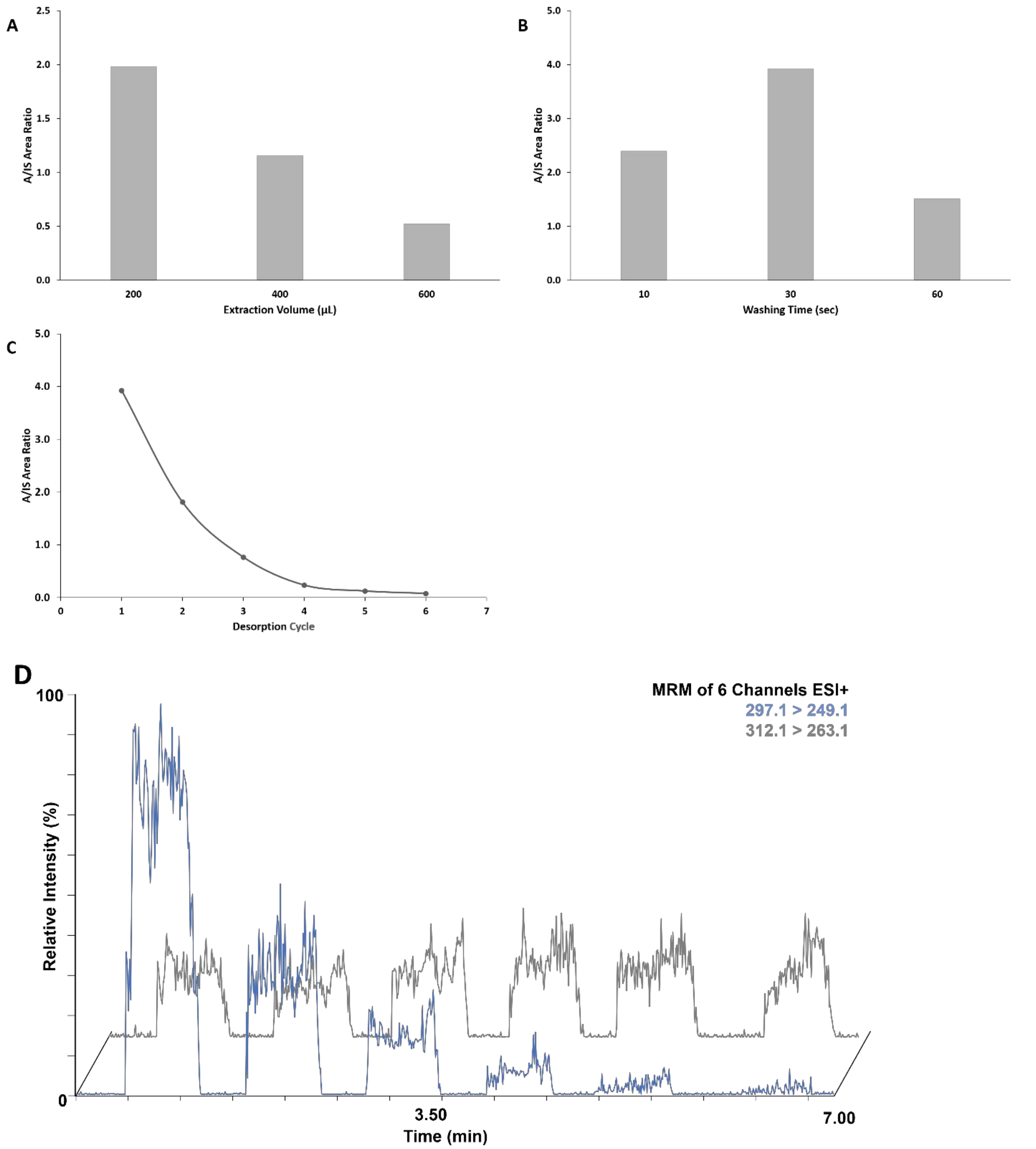

Figure S2. iBS-MS/MS method development. (A) Extraction by immuno-capturing – extraction volume optimization (B) Washing time optimization (C) multi-step desorption assessment and (D) respective representative overlay chronogram of *m/z* 297.1 > 249.1 and *m/z* 312.1>263.1. For detailed conditions and procedures, see text.
